# Naturally-aged microglia exhibit phagocytic dysfunction accompanied by gene expression changes reflective of underlying neurologic disease

**DOI:** 10.1038/s41598-022-21920-y

**Published:** 2022-11-14

**Authors:** Alyssa L. Thomas, Maria A. Lehn, Edith M. Janssen, David A. Hildeman, Claire A. Chougnet

**Affiliations:** 1 Department of Pediatrics, University of Cincinnati College of Medicine, Cincinnati, OH USA; 2 Division of Immunobiology of Cincinnati Children’s Hospital Medical Center, Cincinnati, OH USA; 3 Immunology Graduate Program, Cincinnati Children’s Hospital Medical Center and the University of Cincinnati College of Medicine, Cincinnati, OH USA; 4 Department of Internal Medicine, Division of Hematology/Oncology, University of Cincinnati College of Medicine, Cincinnati, OH USA; 5 Janssen Research and Development, Spring House, PA 19477 USA

**Keywords:** Alzheimer's disease, Neuroimmunology

## Abstract

Age-associated microglial dysfunction contributes to the accumulation of amyloid-β (Aβ) plaques in Alzheimer’s disease. Although several studies have shown age-related declines in the phagocytic capacity of myeloid cells, relatively few have examined phagocytosis of normally aged microglia. Furthermore, much of the existing data on aging microglial function have been generated in accelerated genetic models of Alzheimer’s disease. Here we found that naturally aged microglia phagocytosed less Aβ over time. To gain a better understanding of such dysfunction, we assessed differences in gene expression between young and old microglia that either did or did not phagocytose Aβ. Young microglia had both phagocytic and neuronal maintenance signatures indicative of normal microglial responses, whereas, old microglia, regardless of phagocytic status, exhibit signs of broad dysfunction reflective of underlying neurologic disease states. We also found downregulation of many phagocytic receptors on old microglia, including TREM2, an Aβ phagocytic receptor. TREM2 protein expression was diminished in old microglia and loss of TREM2^+^ microglia was correlated with impaired Aβ uptake, suggesting a mechanism for phagocytic dysfunction in old microglia. Combined, our work reveals that normally aged microglia have broad changes in gene expression, including defects in Aβ phagocytosis that likely underlies the progression to neurologic disease.

## Introduction

Alzheimer’s disease is partially characterized by the accumulation of amyloid-β (Aβ) plaques in the brain that impair cognitive function^1,2^. Aβ plaque accumulation increases with age and interestingly, correlates with known deficiencies in immune cell function. Although Aβ is normally produced in the brain, its accumulation is prevented by the brain’s resident phagocytic cell, the microglia^3^. In fact, phagocytic deficiencies in microglia are strongly linked to Alzheimer’s disease progression^4–6^. Thus, microglia are central regulators of Aβ homeostasis, and in doing so, act as gatekeepers to Alzheimer’s disease. However, although greater than 95% of cases are considered age-related sporadic Alzheimer’s disease and the likelihood of developing Alzheimer’s disease exponentially increases after the age of 65^7^, many of the studies linking phagocytic deficiencies in Alzheimer’s disease have employed transgenic mouse models that overproduce Aβ, overwhelm microglial phagocytic capacity, and artificially accelerate disease development^8^. The few studies that have examined microglial function in normally aged mice reported defects in phagocytic dysfunction, including phagocytosis of Aβ as early as 12 months of age^9,10^. However, as 12-month-old mice represent middle aged humans, the microglial phagocytosis of Aβ in old mice (> 16 months-old) remains unknown.

Here, we investigated the role normal aging plays on microglial phagocytic function. Consistent with reported deficiencies in phagocytic function of other myeloid cells^11–14^, we found that both the frequency and per cell phagocytic capacity were reduced in aged microglia. Mechanistically, aged microglia had decreased expression of several phagocytic receptors including triggering receptor expressed on myeloid cells 2 (TREM2) which correlated with decreased ability to phagocytose Aβ. Further, bulk mRNAseq analysis revealed that young microglia displayed increase expression of genes important in phagocytosis, as well as neuronal maintenance whereas aged microglia, regardless of phagocytosis status, had increased expression of gene associated with proliferation.

## Materials and methods

### Mice

Young (≤ 3 months) and aged (≥ 18 months) male and female C57Bl/6 mice were used for all experiments. Aged mice were obtained from the National Institute of Aging (NIA) colony located at Charles River Laboratories (Wilmington, MA) and young mice were bred in house or obtained from the NIA. Mice obtained from NIA were allowed to acclimate to our mouse facility for at least one week before experimentation. All animal procedures were performed in accordance with relevant guidelines and regulations, including the Animal Research: Reporting in Vivo Experiments (ARRIVE) guidelines and were reviewed and approved by the Institutional Animal Care and Use Committee at the Cincinnati Children’s Hospital Research Foundation (IACUC 2019-0049).

### Microglial enrichment

The protocol for enrichment of immune cells was adapted from^15^. Briefly, immediately after euthanasia by CO_2_ inhalation, mice were perfused by inserting at 27G butterfly needle into the apex of the heart’s left ventricle, incising the right atrium, and pumping ~ 30 mL of cold sterile PBS through the mouse until the liver paled. After perfusion, the brains were harvested from the mice and manually dissociated through a 100 µm cell strainer to create a single cell suspension. Next, the samples were further digested by incubating with 2 U/mL of Liberase with low thermolysin concentration (Roche Diagnostics) for 1 h. After the digestion incubation, the cell suspension was sieved through a 70 µm cell strainer and rinsed thoroughly with HBSS containing 666 U/ml DNAse I (Roche Diagnostics). After pelleting cells, myelin and cellular debris was removed by resuspending in a 25% Percoll Plus (GE Healthcare) density gradient medium where immune cells pelleted after centrifugation (521 × g for 20 min).

### Aβ42 peptide phagocytosis assay and flow cytometry

Amyloid Beta 1-42 peptide (Aβ42) conjugated to HiLyte 488 (Anaspec, Fremont, CA) was initially resuspended and fibrilized as reported in^16,17^. Briefly, Aβ42 was resuspend in 10 nM NaOH (10% of final volume) and then the concentration was adjusted to 1 mg/ml using PBS. Resuspended Aβ42 was frozen at − 20 °C until use. Aβ42 was thawed and incubated at 37 °C overnight (12–14 h) to fibrilize prior to the phagocytosis assay. Aβ42 was added to culture with either young or aged enriched microglia. Duration of culture is noted in each figure. After microglial enrichment and/or phagocytosis assay, samples were stained for flow cytometry using a fixable LiveDead viability stain (ThermoFisher Scientific) and surface stained with the following antibodies: anti-CD45 (clone: 104), -CD11b (clone: M1/70) (Biolegend), and -TREM2 (clone: 237920) (R&D Systems). Aβ42 uptake was evidenced by the presence of HiLyte 488 in microglia (CD45 intermediate and CD11b^+^ cells) and was quantified via flow cytometry. Data was acquired on a FACSCanto flow cytometer from BD Biosciences and analyzed using FlowJo software (Ashland, OR) or an imaging flow cytometer (Amnis ImageStreamX Mark II, Luminex).

### Aβ42^+/−^ microglia flow sorting, RNA extraction, and RNA sequencing

After microglial enrichment and phagocytosis of Aβ42 peptide, brain cells from 4 young to 4 old mice were stained for flow sorting using fixable LiveDead viability stain (ThermoFisher Scientific) and CD45 and CD11b antibodies (Biolegend). Young and old microglia (live, CD45^int^, CD11b^+^) were sorted into two groups: Aβ42 positive and Aβ42 negative microglia using a FACSAria II Cell Sorter (BD Biosciences). Directional polyA RNA-seq was performed by the Genomics, Epigenomics and Sequencing Core at the University of Cincinnati using established protocols as previously described^18,19^. Briefly, total RNA quality was assessed by Bioanalyzer (Agilent, Santa Clara, CA). Using good quality total RNA as input, polyA RNA was isolated using NEBNext Poly (A) mRNA Magnetic Isolation Module (New England BioLabs, Ipswich, MA). Enrichment of the polyA RNA using SMARTer Apollo automated NGS library prep system (Takara Bio USA, Mountain View, CA) and concentration of the polyA RNA to 6 ul via CentriVap micro IR Vacuum Concentrator (Labconco, Kansas City, MO) was performed prior to library preparation. Next, libraries were prepared using NEBNext Ultra II Directional RNA Library Prep kit (New England BioLabs). After verifying library QC and quantification via real-time qPCR (NEBNext Library Quant Kit, New England BioLabs), individually indexed libraries were proportionally pooled and sequenced using NextSeq 550 sequencer (Illumina, San Diego, CA) using sequencing setting of single read 1 × 85 bp. After sequencing, Fastq files were automatically generated by Illumina BaseSpace Sequence Hub for downstream data analysis.

### Differential gene expression analysis

RNA-seq reads in FASTQ format were first subjected to quality control to assess the need for trimming of adapter sequences or bad quality segments. The programs used in these steps were FastQC v0.11.5^20^, Trim Galore! v0.4.2^21^, and cutadapt v1.9.1^22^. The trimmed reads were aligned to the reference mouse genome version mm10 with the program STAR v2.5.2a^23^. Aligned reads were stripped of duplicate reads with picard tools v1.89^24^. Gene-level expression was assessed by counting features for each gene, as defined in the NCBI’s RefSeq database^25^. Read counting was done with the program feature Counts v1.6.2 from the Rsubread package^26^. Raw counts were normalized as transcripts per million (TPM). Differential gene expressions between groups of samples were assessed with the R package DESeq2 v1.26.0^27^. Plots were generated using the ggplot2^28^ package and base graphics in R. Pathway analysis was performed by using ToppGene Suite^29^ where differential expressed genes that had a* p* value of ≤ 0.1 and a log2 (Fold Change) ≥ 0.5 were used as the input.

### Statistics

Statistical analysis was performed using GraphPad Prism. Data are presented as ± standard error of mean (SEM). Significance was tested with Student’s t test for differences between two groups. For line graphs, line of best fit equations were determined for each line and compared using simple linear regression. The* p* values of < 0.05 were considered to be significant and are indicated with the following nomenclature: * *p* < 0.05.

## Results and discussion

### Loss of phagocytic capacity in microglia from aged mice

**Figure 1:**
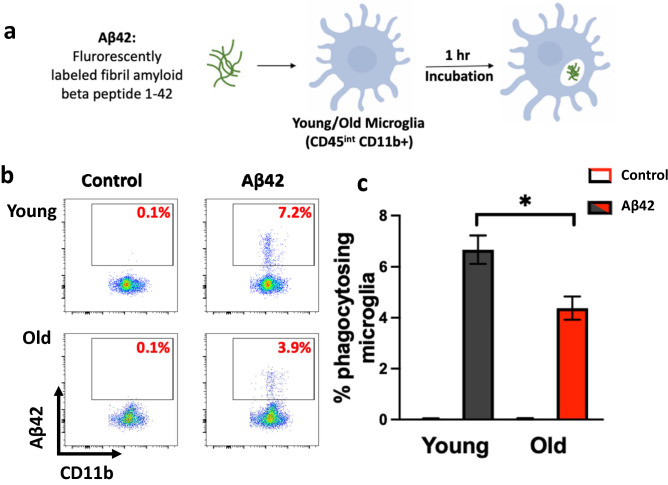
Aged microglia have reduced phagocytic capacity. Brains from female young (2 months) and old (19 months) mice (n = 3) were processed and live CD45 intermediate CD11b^+^ cells (microglia) were assessed for phagocytic capacity. (**a**) Cells were incubated with 0.5 μM of fluorescent fibril Aβ42 or medium (control) for 1 h and phagocytosis was assessed by flow cytometry, images made using ©BioRender - biorender.com. (**b**–**c**) Representative flow plots of 1 replicate of 4 are shown and mean ± s.e.m % of microglia showing phagocytosis of fluorescent Aβ42 are graphed *p ≤ 0.05, Student’s *t* Test.

One of the main functions of microglia is their ability to recognize and phagocytose cellular debris including neurotoxic molecules such as Aβ, whose accumulation can impede cognitive function leading to Alzheimer’s disease^30^. To determine the effect of normal aging on microglial phagocytic function, we assessed phagocytic uptake of Aβ in primary microglia (identified as CD45^int^ and CD11b^+^, Suppl. Figure 1) from young and aged mice. Immune cells were isolated from the brains of young (≤ 3 months) and aged (≥ 18 months) C57Bl/6 mice, cultured with or without fibrilized fluorescently conjugated amyloid-β 1-42 (Aβ42) for 1 h, and phagocytosis was assessed by flow cytometry (Fig. 1a). While the overall frequency of microglia was not altered in aged mice, the frequency of microglia that phagocytosed Aβ42 was significantly reduced in aged mice compared to their younger counterparts (Fig. 1b,c). As flow cytometry is unable to distinguish cell surface binding of Aβ42 versus phagocytosis/internalization, we utilized imaging flow cytometry to confirm Aβ internalization by microglia (Suppl. Figure 2). Further, phagocytic differences between young and old microglia were not due to binding differences as assessed by the lack of significant difference in the percentage of Aβ42^+^ microglia at 4 °C (Suppl. Figure 3). Additionally, while it is possible that these phagocytic differences are due to lysosomal degradation differences between young and aged microglia, studies of Aβ degradation largely examine degradation at much later timepoints (24–48 h)^31–33^ suggesting enhanced degradation would not likely be the major mechanism underlying decreased percentage of Aβ42^+^ microglia in aged mice.

**Figure 2:**
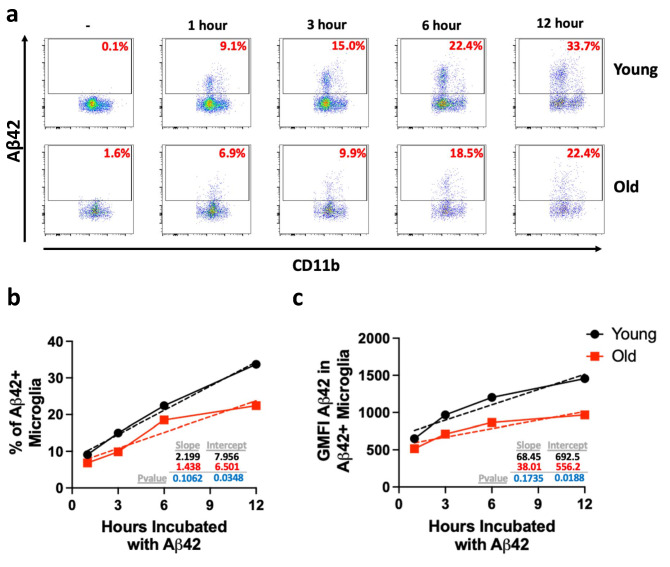
Aged microglia have less uptake capacity of Aβ overtime than their younger counterparts. Microglia from 3 young (2 months) or old (22 months) female mice were isolated, combined with their age-matched counterparts, and divided into 5 time points where they were incubated with 2.5 μM of Aβ42 for 1, 3, 6, and 12 h. Microglia were analyzed via flow cytometry to measure phagocytosis. (**a**) Representative flow plots of 1 replicate showing uptake of Aβ42 by microglia over time. (**b**) % of microglia that are Aβ42 positive over time. Dashed lines indicate line of best fit. (**c**) Geometric mean of Aβ42 in Aβ42^+^ microglia. Dashed lines indicate line of best fit. Simple linear regression was used to determine differences between the lines of best fit.

Next, to determine whether the defect in in vitro Aβ phagocytosis was observed at later timepoints, we assessed Aβ42 uptake via flow cytometry at 1, 3, 6, and 12 h after incubation. Although the percentage of Aβ42^+^ young and old microglia steadily increased over time, the percentage of old Aβ42^+^ microglia continued to trail that of young microglia (Fig. 2a,b). Additionally, the amount of phagocytosed Aβ42, as measured by the fluorescence intensity per cell (geometric mean of fluorescence, gMFI), in old microglia was lower than in their younger counterparts, reaching their phagocytic maximum by 12 h compared to young microglia whose gMFI continued to increase (Fig. 2c). These data show that aged mice have fewer microglia capable of Aβ42 phagocytosis and, on a per cell basis, phagocytose less Aβ42 than those from young mice.

### RNAseq analysis reveals pervasive aged microglial dysfunction

**Figure 3:**
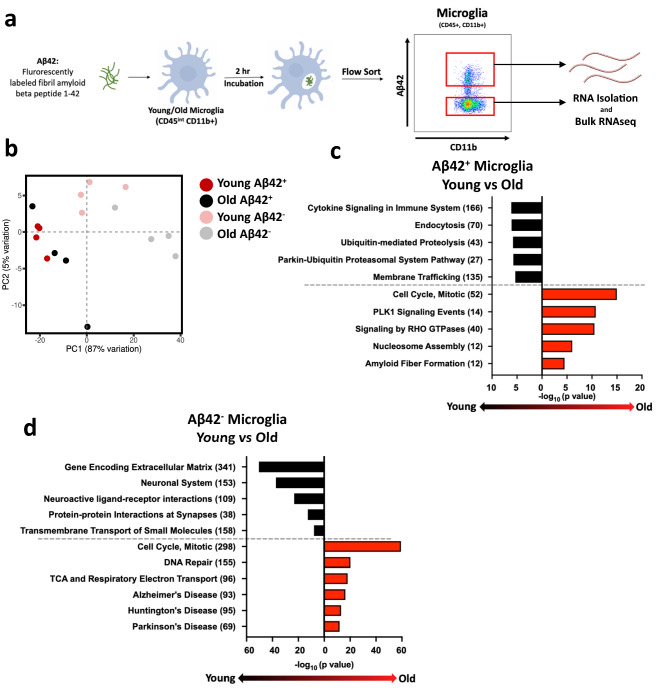
Pathway analysis of young and old microglia that either did or did not take up peptide showed deficiencies of old microglia. Young (2 months) and old (18 months) microglia (3 male mice were combined to make one replicate; this was repeated for a total of 4 replicates/group) were incubated with 2.5 μM of Aβ42 for 2 h and then were FACSorted based on Aβ42 uptake. Sorted microglia’s RNA was isolated and used to run bulk RNAseq analysis. (**a**) Schematic of phagocytosis assay and flow sorting, images made using ©BioRender - biorender.com. (**b**) Principal component analysis plot of the four different microglia groups (Young Aβ42^+^, Young Aβ42^−^, Old Aβ42^+^, Old Aβ42^−^) with 4 replicates each. Pathway analysis of DEGs in both the young and old (**c**) Aβ42^+^ and (**d**) Aβ42^−^ microglia graphed based on their p value. The number of DEGs in each pathway are indicated in parentheses.

Given that the frequency and capacity of aged microglia to phagocytose Aβ was reduced, we took an unbiased approach to understand gene expression changes in aged microglia that could contribute to their defective phagocytosis. While other studies examined differences between young and old at steady-state^34,35^, we cultured aged and young microglia with fluorescent Aβ42, FACs sorted microglia that did (Aβ42^+^) and did not (Aβ42^−^) phagocytose and we subjected them to bulk mRNAseq (Fig. 3a) to determine age-associated differences in microglia based off their phagocytosing status. We believe this comparison provides a more physiologically relevant scenario as transient interactions with Aβ could be occurring with all microglia and thus could be accounted for using this strategy. To determine the relationships between these sorted populations, we performed principal component analysis (PCA). PCA showed that phagocytosing (Aβ42^+^) young and old microglia clustered together suggesting that they had very similar gene expression (Fig. 3b, compare dark red circles to black circles). In contrast, young and old microglia that did not phagocytose (Aβ42^−^) cluster distantly from those that did phagocytose (Aβ42^+^). Further, aged microglia that did not phagocytose Aβ42 clustered further away from all other subsets (Fig. 3b, compare gray circles to all other circles). Together, these data show that age-associated changes in gene expression are associated with microglial phagocytic dysfunction.

To gain insight into the biology underlying defective phagocytosis with age, we generated lists of differentially expressed genes (DEGs) and performed pathway analysis. In those microglia that phagocytosed Aβ42, there were 3402 genes that were upregulated in young compared to old microglia and 856 genes that were increased in old compared to young microglia (Table 1). Pathway analysis showed that young microglia had increased expression of genes involved in endocytosis, membrane trafficking genes, and proteolysis (Fig. 3c). Notably, relative to old microglia, young microglia showed increased expression of RAB and ARF genes (Suppl. Figure 4a) which are known to be involved in endosomal trafficking, vesicle formation, and intracellular transport^36^. Given that defective function of both of these families of small Ras GTPases have been implicated in neurodegenerative diseases^37^, it is possible that their decreased expression could contribute to reduced microglial phagocytic capacity and contribute to disease in aged microglia.

**Table 1:** Number of genes upregulated in young and old microglia that did and did not phagocytose Aβ42.

	Up in young	Up in old
Aβ42^+^	3403	857
Aβ42^−^	5245	5539

Following phagocytosis, ingested material is broken down into reusable substrates. Interestingly, young microglia had increased expression of genes associated with proteasomal subunits (*Psmd1*, *Psmd3*, *Psmd11*, *Psmd12*, and *Psmd14*) as well as immunoproteasome subunits (*Psmc6* and *Psmd7*) (Suppl. Figure 4b). This data is consistent with prior work suggesting decreased proteasomal processing in aged, senescent cells^38^. Further, young microglia that phagocytosed Aβ42 had increased expression of several cytokine and cytokine receptor genes relative to old microglia. Increased mRNA levels of *Il1a*, *Il18*, *Il12b*, *Ifng*, and *Csf1* in the young microglia indicated a robust pro-inflammatory profile (Fig. 3c, Suppl. Figure 4c). Furthermore, cytokine receptors such as *Il1rl2*, *Il12rb2*, *Il6ra*, *Il10ra*, and *Csf1r* (Suppl. Figure 4c) and genes involved in cytokine signaling including, TRAF, SMAD, and NFKB genes (Suppl. Figure 4d), were also highly expressed likely enabling these young microglia to respond to multiple cytokines. Overall, the increase in immune cell activation genes show that young phagocytosing microglia are capable and prepared to respond to immunological signals and targets. We acknowledge that upregulation of pro-inflammatory markers in the young microglial compartment compared to old microglia is not in agreement with previous studies that reported increased inflammatory signatures in other aging cells such as macrophages and dendritic cells^39,40^. However, a recent single cell RNAseq study showed that only a small proportion of aging microglia have an inflammatory signature^34^, suggesting that microglia are not the primary source of inflammaging in the brain. Although we note that our bulk mRNAseq approach would not reveal subtle trends present only in subpopulations and can only unveil the pathways present in most microglia.

**Figure 4:**
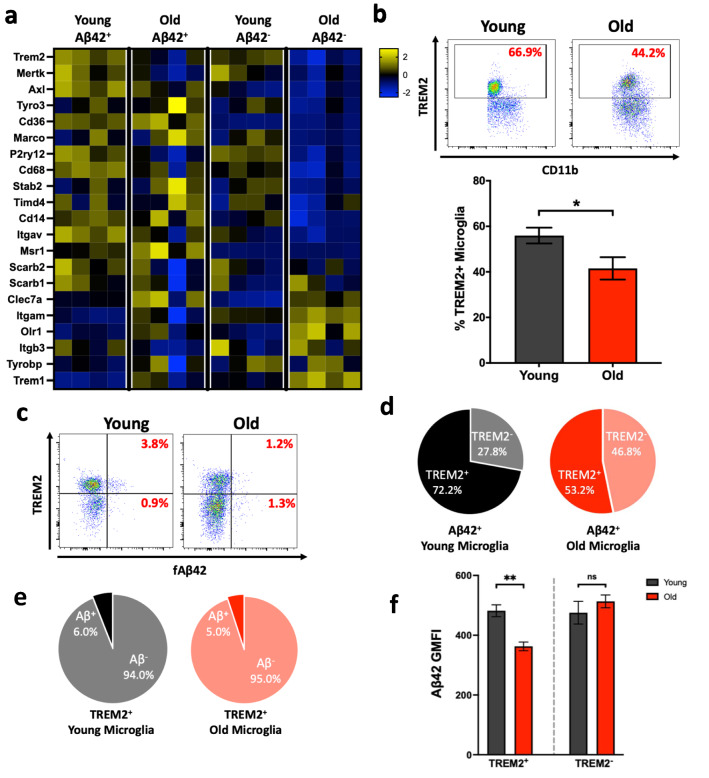
Expression of TREM2, an Aβ42 receptor, is decreased in aging microglia. (**a**) Heatmap representation of phagocytic receptor gene expression levels for Young Aβ42^+^, Young Aβ42^−^, Old Aβ42^+^, and Old Aβ42^−^. The scale represents the row Z-score from 2 (highest expression) to − 2 (lowest expression). Young (2 months) and aged (21 months) microglia were incubated with 0.5 μM Aβ42 for 1 h and stained for microglial markers and TREM2 (n = 3/group; replicated 4 times with both male and female mice) (**b**) Representative flow plots (n = 3/group) of young and aged microglia TREM2 expression and graph of % of young and old microglia that are TREM2^+^. (**c**) Representative flow plots of young and aged microglia TREM2 expression and Aβ42 uptake. (**d**) Graphs showing the average percentage of TREM2 expression in young (black) and old (red) Aβ42^+^ microglia and (**e**) Average percentage of Aβ fluorescence in young (black) and old (red) TREM2^+^ microglia. (**f**) Geometric mean fluorescence of Aβ42 in TREM2^+^ and TREM2^−^ Aβ42^+^ microglia. *p ≤ 0.05, **p ≤ 0.01, mean ± s.e.m, Student’s *t* Test.

In contrast to the young phagocytosing microglia, pathway analysis of young microglia that did not phagocytose Aβ42 revealed increased expression of genes associated with extracellular matrix and synaptic interaction (Table 1). Young microglia had significantly increased expression of several matrix metallopeptidases (*Mmp3*, *Mmp11*, *Mmp12*, *Mmp15*, *Mmp16*) and ADAM metallopeptidases (*Adamts4, Adamts3, Adamts2, Adam23, Adam7*) (Fig. 4d, Suppl. Figure 4e). Recent studies have reported that upregulation of ECM correlates with remodeling of neuron synapses^41,42^, thus suggesting that these young non-phagocytosing microglia may play a very different role than their phagocytosing counterparts. Notably, microglia are important for remodeling and maintenance of neuronal synapses and increased expression of these metallopeptidases is likely critical for this process^41,43,44^. Neuronal-type pathways, such as “neuronal system”, “neuroactive ligand-receptor interactions”, and “protein–protein interactions at synapses”, were also notably increased in these young non-phagocytosing microglia (Fig. 3d), suggesting that non-phagocytosing microglia are programmed more for neuronal remodeling rather than immune function. This bifurcated functionality of young microglia is in agreement with the concept of heterogeneity of the microglial compartment, an expanding area of interest in neuronal research^45,46^. These data suggest that there are microglia predisposed to respond to damage signals like Aβ that can be activated very quickly after recognition of these signals, while other microglial populations are mainly involved in neuronal remodeling and maintenance.

Pathway analysis of old phagocytosing microglia indicates a very different cellular state from that of their young counterparts. Cell cycling and associated pathways dominated as the top pathways in these cells (Fig. 3c). Cyclins and cyclin-dependent genes (*Ccnb1*, *Ccnb2, Cdk1, Cdkn2c*), cell division cycle genes (*Cdca5*, *Cdc20*, *Cdc25b*, *Cdca8*) and nucleosome assembly genes (*Cenpn*, *Cenpk*, *Cenpl)* were notably upregulated in the old phagocytosing microglia. Interestingly, increased proliferative capacity also appears to be a defining factor in old microglia that did not phagocytose (Fig. 3d, Suppl. Figure 4f,g ), along with upregulation of DNA repair genes. Such unregulated cell cycling could be damaging to the microglia. Indeed, increased microglial proliferation is an early characteristic of disease-associated microglia (DAMs)^47^, which have been reported in multiple mouse models of neurodegenerative diseases such as Alzheimer’s disease^48^, prion disease^49^, and ischemic stroke^50^. Further, a recent study even suggests that senescent replication promotes the emergence of DAMs^51^. Thus, old microglia may be displaying early signs of dysfunction that mirror microglia in a diseased state. Due to the deficiencies in aged microglia, this increased cell cycling may be the attempt of microglial precursors to fill the open niche left by these deficiencies. One intriguing hypothesis may be that Aβ acts like a homeostatic regulator of microglial proliferation. Thus, the increased Aβ seen in an aging brain encourages the production of more microglia to combat further accumulation. However, despite this increase in proliferation, we did not see increases in overall percentage of microglia in aged mice. This may be due to proliferative stress, indicated by upregulation of DNA repair pathways and PLK1 signaling, in aged microglia. Thus, aged cycling microglia are likely to be prone to cell death that prevents increased microglia numbers with age. Additionally, such increased cell cycling may place intense energetic demands on the aged cells, a concept supported by the fact we also found upregulated expression of metabolic-related genes implicated in the citric acid cycle (TCA) and the electron transport chain (ETC) (Fig. 3d).

Beyond a proliferative phenotype, pathway analysis of old microglia that did not phagocytose also revealed pathways associated with neurodegenerative diseases, such as *App*, *Adam17*, *Psen1*, and *Psen*2^52,53^(Suppl Figure 4h). Additionally, old microglia that did phagocytose had an upregulation of the amyloid fiber formation pathway (Fig. 3c) further hinting those old microglia, regardless of their phagocytic status, exhibit tell-tale signs of microglial dysfunction and disease state.

### Decreased TREM2 expression in aged microglia correlate with phagocytic uptake

Given the dysfunctional phagocytosis in aged microglia, we next examined the differences in gene expression of over 20 receptors potentially involved in microglial phagocytosis. Old microglia that did not phagocytose Aβ42 had low expression of two-thirds of these genes. Expression of these genes encoding these receptors was decreased compared to not only their younger non-phagocytosing counterparts but also to old microglia that did phagocytose (Fig. 4a), suggesting that decreases in phagocytic capacity may be due to a population of old microglia losing their ability to recognize phagocytic material. Interestingly, we found that mRNA expression of one phagocytic receptor, TREM2, was particularly low. Studies have shown that TREM2 directly binds Aβ^54^, is a vital component of Aβ clearance^55^, and has been linked to Alzheimer’s disease in both Alzheimer’s patients^56,57^ and in mouse models^58,59^. Many studies concluded that TREM2 expression is positively correlated with uptake of Aβ^17,60^ and loss of TREM2 function leads to Aβ accumulation^61^, although a recent study found that TREM2 deficiency increased phagocytosis in an Alzheimer’s disease mouse model^62^. Overall, the literature suggests that normal age-related declines in TREM2 expression may contribute to defective phagocytosis in aged microglia.

To confirm these differences in TREM2 mRNA levels, we next assessed protein levels of TREM2 on young and old microglia by flow cytometry. Aged mice had an overall decrease in the frequency of TREM2^+^ microglia compared to their younger counterparts (Fig. 4b), consistent with prior studies that showed decreased *Trem2* mRNA expression in aged microglia^35^. We next asked whether TREM2^+^ cells were the predominant microglial population involved in Aβ phagocytosis. In young mice, nearly 75% of Aβ42^+^ microglia expressed TREM2, while in aged mice, only 50% of aged Aβ42^+^ microglia were TREM2^+^ (Fig. 4c,d). Although the frequency of TREM2^+^ microglia that were Aβ42^+^ was similar in young and aged mice (Fig. 4e), aged TREM2^+^ Aβ42^+^ microglia phagocytosed less Aβ as shown by decreased gMFI (Fig. 4f). These data suggest that old TREM2^+^ microglia are still capable of taking up Aβ42, but that other receptors, which may also be defective, contribute to Aβ42 phagocytosis in aged microglia. Combined, these data suggest that the decreased proportion of TREM2^+^ old microglia contribute to the overall decreased ability to phagocytose Aβ peptide, which might contribute to Aβ accrual during late-onset Alzheimer’s disease.

In summary, our data show that microglia from normally aged mice exhibit many tell-tale signs of dysfunction, including deficits in phagocytosis of Aβ peptide and substantially altered expression of genes involved in cell cycling, synaptic pruning, and neuronal homeostasis. Importantly, much of the existing data on microglial function in aging have been generated in accelerated genetic models of Alzheimer’s disease^63–65^ which may not reflect normal progressive inflammatory environment that develops with age. Our study highlights the importance of further research on age-related dysfunctions of microglia and how they contribute to driving age-associated sporadic disease.

## Supplementary Information

Supplementary Information.

## Data Availability

The RNAseq dataset generated and analyzed in this current study is available in the Gene Expression Omnibus (GEO) repository (Accession # GSE205803).
