# Supplementary material for: Naturally-aged microglia exhibit phagocytic dysfunction accompanied by gene expression changes reflective of underlying neurologic disease
Source: Sci Rep. 2022 Nov 14;12:19471. doi: 10.1038/s41598-022-21920-y (PMC9663419; doi:10.1038/s41598-022-21920-y)
Supplement: Supplementary file 1 — Supplementary Information. [file 41598_2022_21920_MOESM1_ESM.pdf]

Naturally-aged microglia exhibit phagocytic dysfunction accompanied by gene expression changes reflective of underlying neurologic disease

Alyssa L. Thomas[1,2,3], Maria A. Lehn[3,4], Edith M. Janssen[3,5], David A. Hildeman[1,2,3*], Claire A. Chougnet[1,2,3*]

[1]Department of Pediatrics, University of Cincinnati College of Medicine, Cincinnati, OH, United States, [2]Division of Immunobiology of Cincinnati Children's Hospital Medical Center, Cincinnati, OH, United States, [3]Immunology Graduate Program, Cincinnati Children's Hospital Medical Center and the University of Cincinnati College of Medicine, Cincinnati, OH, United States
[4] Department of Internal Medicine, Division of Hematology/Oncology, University of Cincinnati College of Medicine, Cincinnati, Ohio, USA
[5]Janssen Research and Development, Spring House, PA 19477
Cincinnati Children's Hospital Medical Center, Cincinnati, OH, United States
* Corresponding Authors

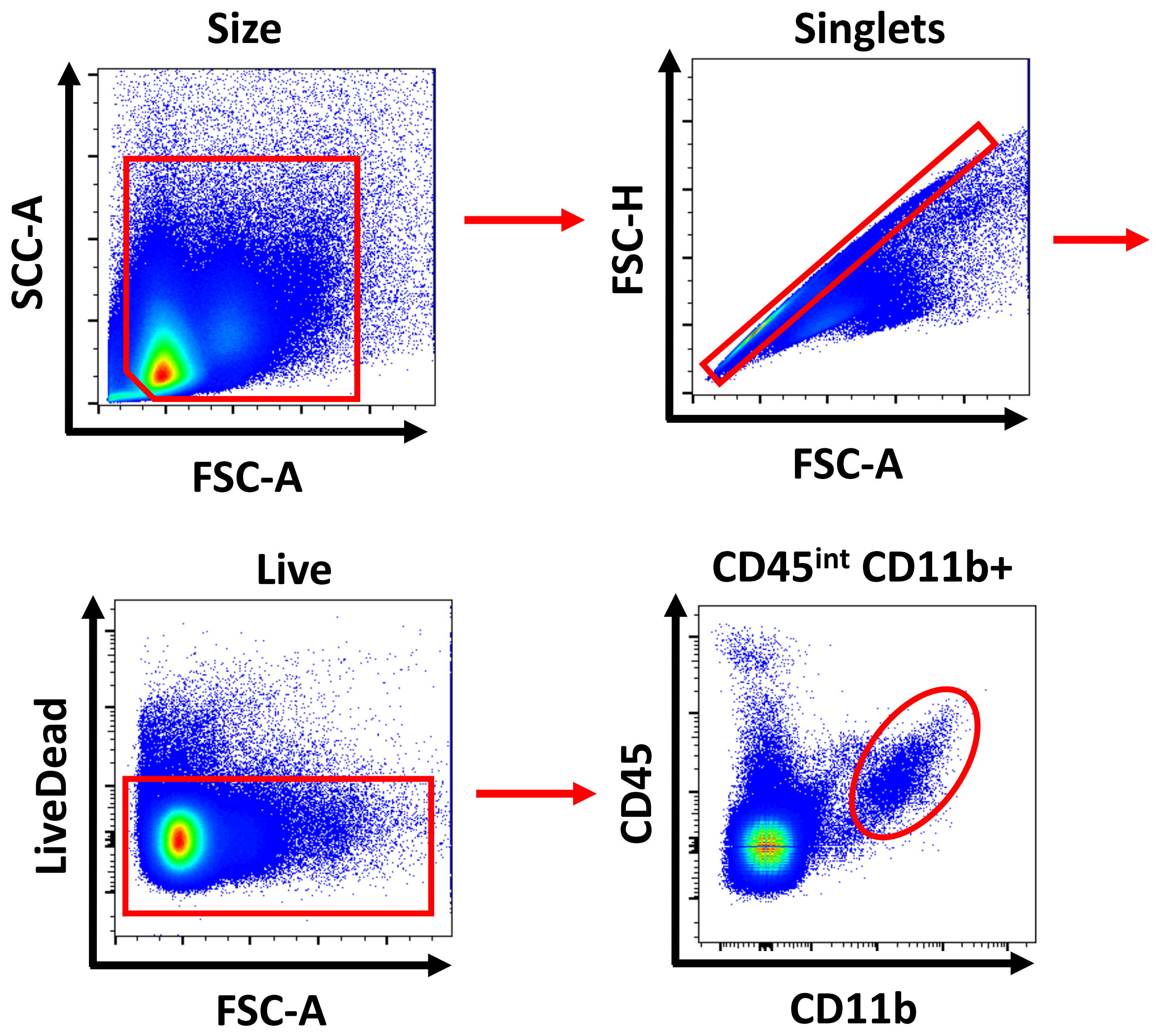

Supplemental Figure 1: Flow cytometry gating strategy to identify microglia after immune cell isolation of the brain. Representative flow cytometry gating of single cell suspension of isolated immune cells from the brain. Microglia were identified as live, $CD45^{int}$ $CD11b^{+}$ cells.

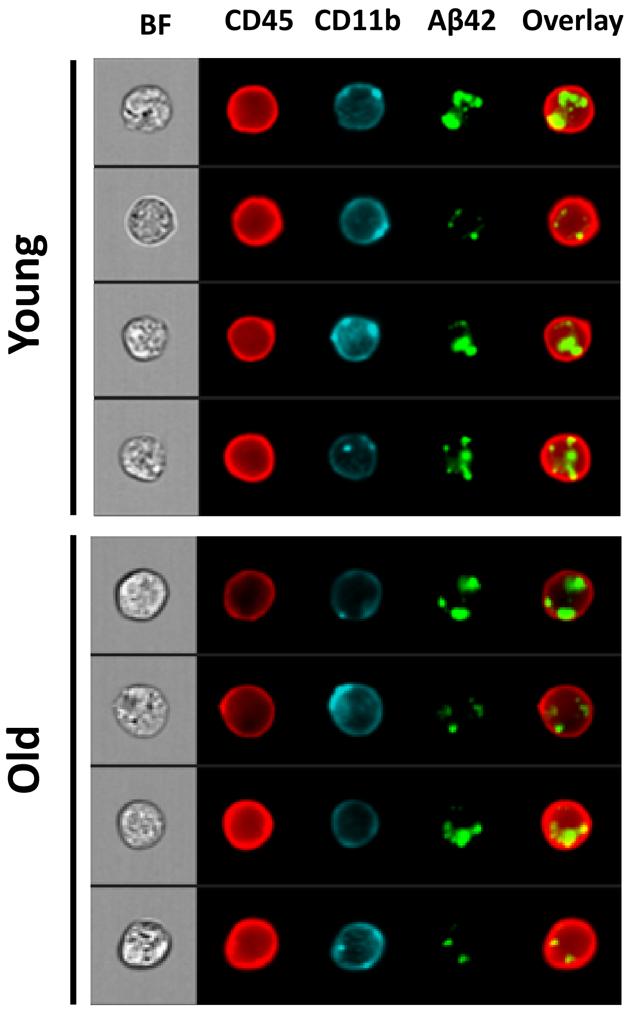

Supplemental Figure 2: Imaging flow cytometry of Aβ42 uptake. Representative imaging flow cytometry images of microglia upon 2hr incubation of Aβ42. BF, brightfield. Red indicates CD45, blue indicates CD11b, and green indicates Aβ42. Original magnification 60X.

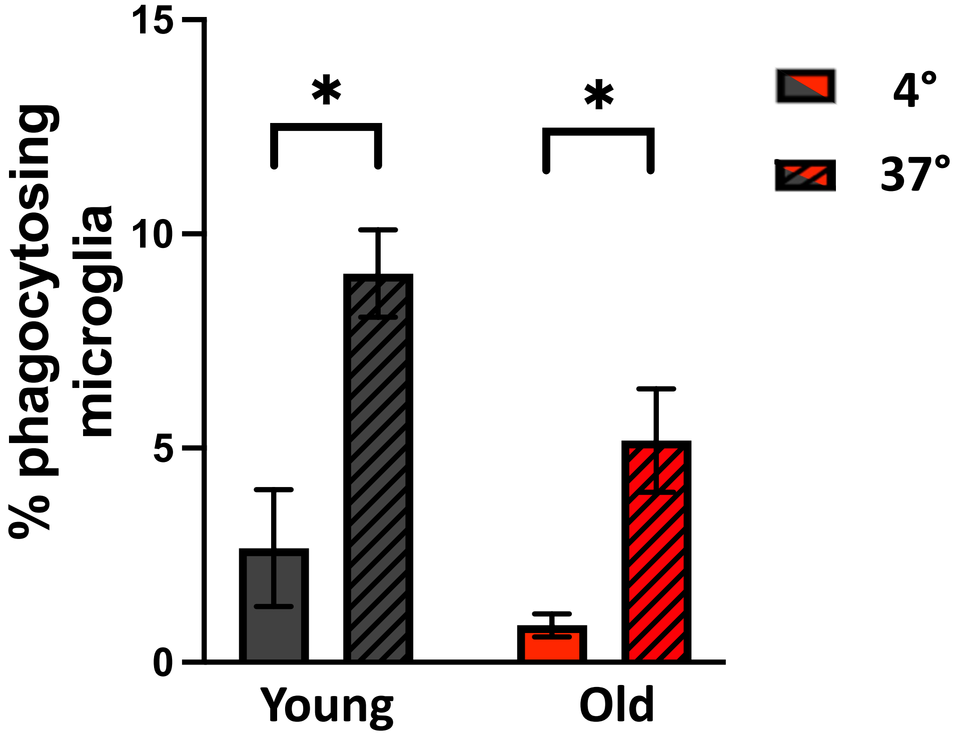

Supplemental Figure 3: Microglial phagocytosis at 4° and 37°. Brains from female young (3 months) and old (22 months) mice (n=3-4) were processed and live CD45 intermediate $CD11b^{+}$ cells (microglia) were assessed for phagocytic capacity. Cells were incubated with 0.5µM of fluorescent fibril Aβ42 or medium (control) for 1 hour and phagocytosis was assessed by flow cytometry. Graphs are shown as mean ± s.e.m % of microglia showing phagocytosis of fluorescent Aβ42 are graphed $*p \leq 0.05$, Student's *t* Test

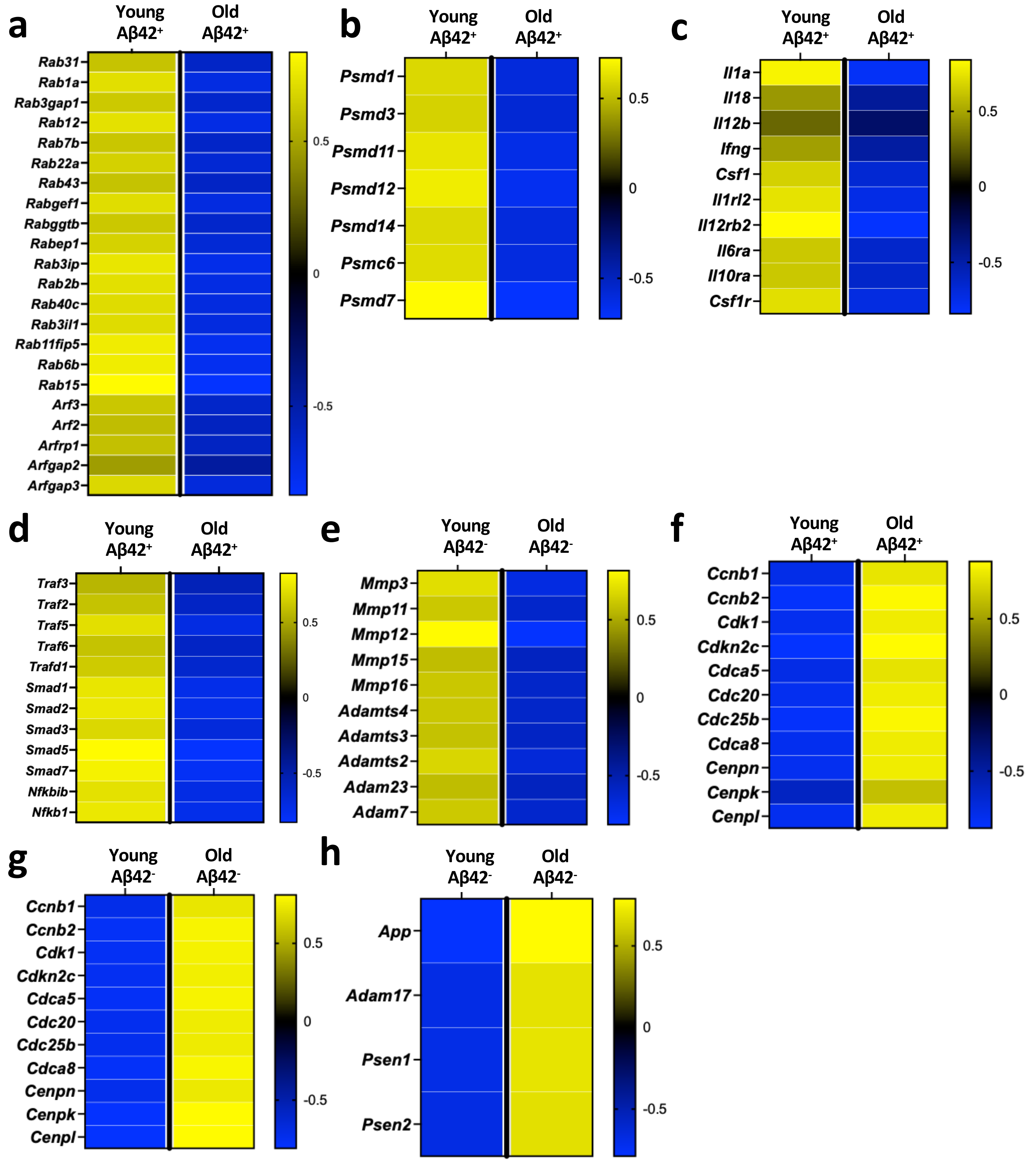

Supplemental Figure 4: Average gene expression of select genes from bulk RNAseq. Heatmap representations of selected genes from bulk RNAseq. Z score comparisons are shown as average gene expression of 4 mice per group. **a), b), c), d), and f)** Heatmaps compare young Aβ42$^+$ microglia to old Aβ42$^+$ microglia. **e), g), and h)** Heatmaps compare young Aβ42$^-$ microglia to old Aβ42$^-$ microglia.
